# Effect of the Diagnosis of Inflammatory Bowel Disease on Risk-Adjusted Mortality in Hospitalized Patients with Acute Myocardial Infarction, Congestive Heart Failure and Pneumonia

**DOI:** 10.1371/journal.pone.0158926

**Published:** 2016-07-18

**Authors:** Eli D. Ehrenpreis, Ying Zhou, Aimee Alexoff, Constantine Melitas

**Affiliations:** Center for the Study of Complex Diseases, NorthShore University HealthSystem, Evaston, Illinois, United States of America; University Hospital Llandough, UNITED KINGDOM

## Abstract

**Introduction:**

Measurement of mortality in patients with acute myocardial infarction (AMI), congestive heart failure (CHF) and pneumonia (PN) is a high priority since these are common reasons for hospitalization. However, mortality in patients with inflammatory bowel disease (IBD) that are hospitalized for these common medical conditions is unknown.

**Methods:**

A retrospective review of the 2005–2011 National Inpatient Sample (NIS), (approximately a 20% sample of discharges from community hospitals) was performed. A dataset for all patients with ICD-9-CM codes for primary diagnosis of acute myocardial infarction, pneumonia or congestive heart failure with a co-diagnosis of IBD, Crohn’s disease (CD) or ulcerative colitis (UC). 1:3 propensity score matching between patients with co-diagnosed disease vs. controls was performed. Continuous variables were compared between IBD and controls. Categorical variables were reported as frequency (percentage) and analyzed by Chi-square tests or Fisher’s exact test for co-diagnosed disease vs. control comparisons. Propensity scores were computed through multivariable logistic regression accounting for demographic and hospital factors. In-hospital mortality between the groups was compared.

**Results:**

Patients with IBD, CD and UC had improved survival after AMI compared to controls. 94/2280 (4.1%) of patients with IBD and AMI died, compared to 251/5460 (5.5%) of controls, p = 0.01. This represents a 25% improved survival in IBD patients that were hospitalized with AMI. There was a 34% improved survival in patients with CD and AMI. There was a trend toward worsening survival in patients with IBD and CHF. Patients with CD and PN had improved survival compared to controls. 87/3362 (2.59%) patients with CD and PN died, compared to 428/10076 (4.25%) of controls, p < .0001. This represents a 39% improved survival in patients with CD that are hospitalized for PN.

**Conclusion:**

IBD confers a survival benefit for patients hospitalized with AMI. A diagnosis of CD benefits survival in patients that are hospitalized with PN.

## Introduction

The Centers for Medicare & Medicaid Services (CMS) and Hospital Quality Alliance (HQA) release quarterly reports on mortality of hospitalized patients with acute myocardial infarction (AMI), congestive heart failure (CHF) and pneumonia (PN) [1–2]. 30-day mortality rates for AMI, CHF, and PN are also reported by CMS. Mortality indicators have been developed by the Agency for Health Research on Quality (AHRQ). The diagnoses of AMI, CHF and PN have become a primary focus of these agencies, since these conditions are common reasons for hospital admission among older adults [3,4]. Reporting of risk-standardized mortality is intended to enhance accountability of collected data by considering patient risk as well as hospital-specific effects (i.e. the risk of mortality for individual hospitals). The overall goal of measuring risk-standardized mortality is advancement of patient outcomes [5–6].It is intuitive that the presence of preexisting co-morbidities may have a negative impact on outcomes in patients that are hospitalized for medical conditions. This effect has been explored in detail in patients with AMI and those undergoing coronary bypass grafting (CABG) by Vaughn, et al [7]. Of interest, their group defined co-morbidities as *non-paradoxical* (preexisting conditions that worsen mortality in hospitalized patients) or *paradoxical* (preexisting conditions that improve mortality in hospitalized patients). Examples of paradoxical co-morbidities found to improve 30 day survival after AMI include hypertension, diabetes, and obesity. There are a variety of potential explanations for the occurrence of paradoxical co-morbidities [8–9].

Prior studies suggest that patients with IBD, particularly Crohn’s Disease (CD), are at higher risk for thromboembolic disorders, coronary artery disease and AMI [10–12]. PN has also been found to occur more commonly in patients with IBD compared to those in the general population [13–14]. However, there has been limited prior evaluation of outcomes, including mortality, in patients with IBD that are hospitalized for these and other common medical conditions. In this study, we analyzed the effect of having a diagnosis of IBD on mortality in patients that were hospitalized for AMI, CHF or PN.

## Methods

### Data Source

A retrospective review of the 2005–2011 National Inpatient Sample (NIS) was conducted. The NIS is the largest publicly available inpatient healthcare database in the United States. It is part of the Healthcare Cost and Utilization Project (HCUP) sponsored by the Agency for Healthcare Research and Quality (AHRQ). The NIS contains approximately a 20% sample of discharges from all community hospitals participating in HCUP, representative of more than 95% of the U.S. population. An estimated seven million hospital admissions per year are reported containing data on primary and secondary diagnoses and procedures, patient demographics, hospital characteristics, length of stay, insurance status, median income by zip code and co-morbidity measures [15]. Information about individual medications is not included in the database.

### Sample Selection

The International Classification of Diseases, 9^th^ Revision Clinical Modification (ICD-9-CM) diagnostic codes was used to identify the study population of interest. The ICD-9-CM is used to code and classify morbidity data from hospitals, physicians’ offices and National Center for Health Statistics (NCHS) surveys [16, 17]. The dataset was created by searching NIS for all patients presenting with ICD-9-CM codes for a primary diagnosis of acute myocardial infarction, pneumonia or congestive heart failure with a co-diagnosis of IBD, CD or UC (Table 1). Data from the NIS does differentiate patients having repeated hospitalizations since each hospital stay is given a unique ID. In-hospital mortality between the groups was compared.

**Table 1:** ICD-9-CM Diagnostic Codes Used to Identify Primary and Co-diagnoses.

Diagnosis	Code
Inflammatory bowel disease (IBD)	569.9
Crohns disease (CD)	555.0, 555.1, 555.2, 555.9
Ulcerative colitis (UC)	556.0, 556.1, 556.2, 556.3, 556.6, 556.8, 556.9
Acute Myocardial Infarction (AMI)	41000, 41001, 41002, 41010, 41011, 41012, 41020, 41021
41022, 41030, 41031, 41032, 41040, 41041, 41042, 41050	
41051, 41052, 41060, 41061, 41062, 41070, 41071, 41072	
41080, 41081, 41082, 41090, 41091, 41092	
Congestive Heart Failure (CHF)	4281, 40291, 4149, 4109, 41189, 41181, 4169, 4150, 42830, 42831
42833, 42832, 40491, 40493, 4281, 4289, 42820, 42821, 42823, 42822	
Pneumonia	486, 4808, 4809, 481, 4821, 48241, 48283, 4829, 485, 4870

### Statistical Analysis

Continuous variables were reported as mean±standard deviation and median (range). The normality assumption for continuous variables was assessed using the Shapiro-Wilk test. Continuous variables were compared between IBD and controls by two-sample T-test or Wilcoxon rank-sum test as appropriate. Categorical variables were reported as frequency (percentage) and were analyzed by Chi-square tests or Fisher’s exact test for co-diagnosed disease vs. control comparisons. Propensity score matching method is widely used in observational studies to reduce selection bias. To fairly compare the outcomes of interests between for co-diagnosed disease group and controls, we conducted the 1:2 for IBD vs. controls or 1:3 for UC and CD vs. controls propensity score matching between patients with co-diagnosed disease vs. controls using greedy 8-to-1 match algorithm [17].Propensity scores were computed by modeling the probability of having the co-diagnosed disease through multivariable logistic regression with following factors: age, gender, race, and total numbers of co-morbidities in records, total numbers of procedures in records, admission type, insurance, income quartiles, hospital beds, hospital control, hospital location, hospital region and hospital teaching status. The matching factors were carefully selected to include all the potential bias and may be take into account in the matching. Comparisons of patient demographics and clinical variables between patients with UC vs. matched controls and patients with CD vs. matched controls are seen in Table A for PN, Table B for AMI and Table C for CHF. These can be found as Tables A-C in the S1 File. Statistical analyses were performed on SAS 9.3 (Cary, NC) Windows platform. A p < 0.05 was considered as statistically significant.

### Ethical considerations

The study was reviewed by NorthShore University HealthSystem Institutional Review Board (IRB) and deemed appropriate for exempt status of IRB oversight due to the de-identified nature of HCUP-NIS data.

### Acute myocardial infarction (Fig 1)

There were 2280 patients with a co-diagnosis of IBD that were admitted for AMI. These were matched with 4560 controls. 94/2280 (4.12%) of patients with IBD and AMI died, compared to 251/4560 (5.5%) of controls, p = 0.01. This represents a 25% improved survival in IBD patients that were hospitalized with AMI. There were 1164 patients with CD admitted for AMI. These were matched with 3494 controls. 47/1164 (4.04%) patients with CD and AMI died, compared to 213/3494 (6.1%) of controls, p = 0.008. This represents a 34% improved survival in IBD patients that were hospitalized with AMI. There were 1123 patients with UC admitted with AMI. These were matched with 3375 controls. 47/1123 (4.19%) patients with UC and AMI died, compared to 207/3375 (6.13%) of controls, p = 0.01, (24% improved survival).

**Fig 1:**
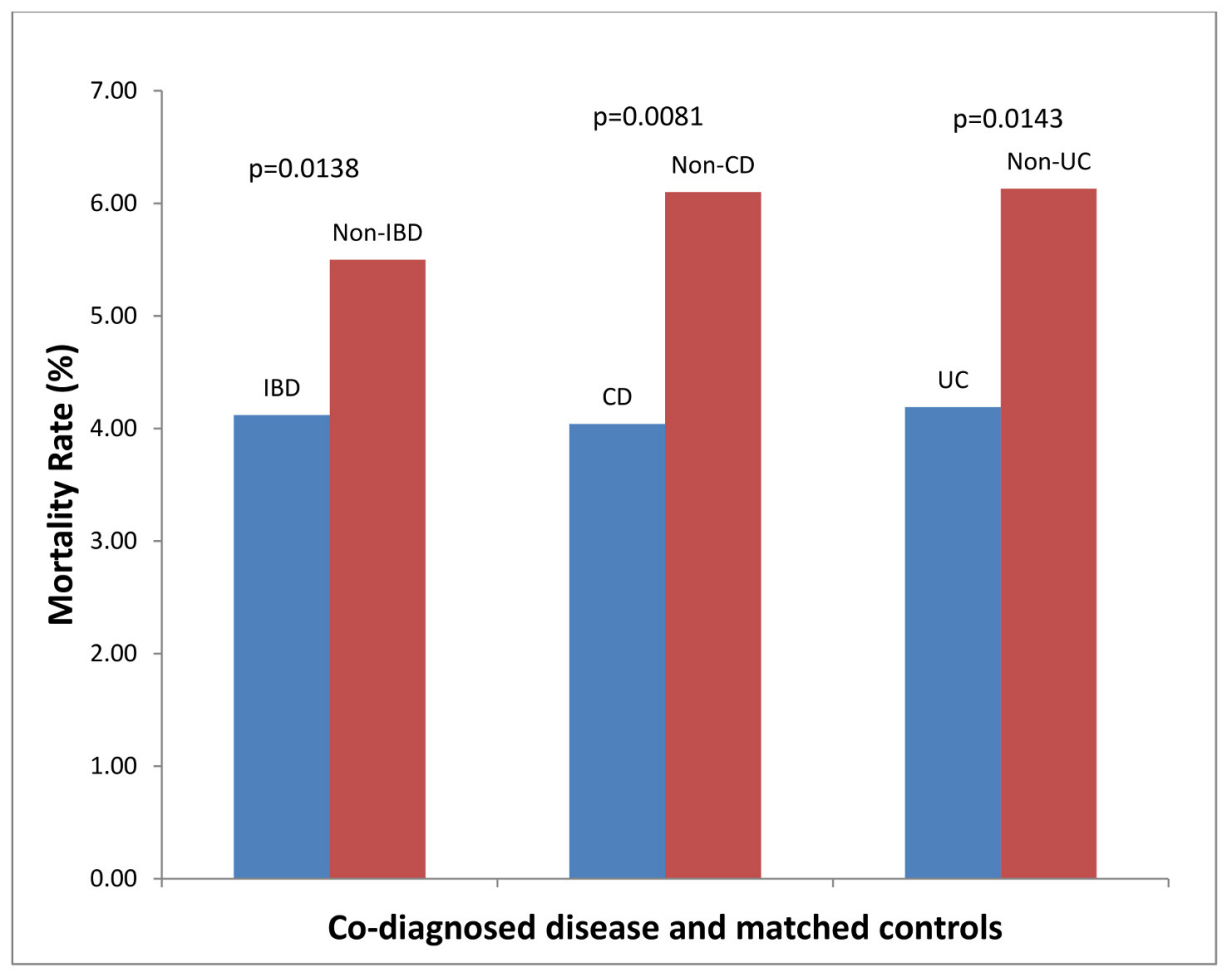
Hospital Mortality Rates for Co-Diagnosis and Matched Controls for Patients Hospitalized with Primary Diagnosis of Acute MI (AMI).

### Congestive heart failure (Fig 2)

There were 1350 patients with a co-diagnosis of IBD that were admitted for CHF. These were matched with 2700 controls. 55/1350 (4.07%) of patients with IBD and CHF died, compared to 94/2700 (3.48%) of controls, p = 0.35. There were 786 patients with CD admitted for CHF. These were matched with 2356 controls. 30/786 (3.82%) patients with CD and CHF died, compared to 68/2356 (2.89%) of controls, p = 0.19. There were 567 patients with UC admitted with CHF. These were matched with 1699 controls. 25/567 (4.41%) patients with UC and CHF died, compared to 48/1699 (2.83%) of controls, p = 0.06.

**Fig 2:**
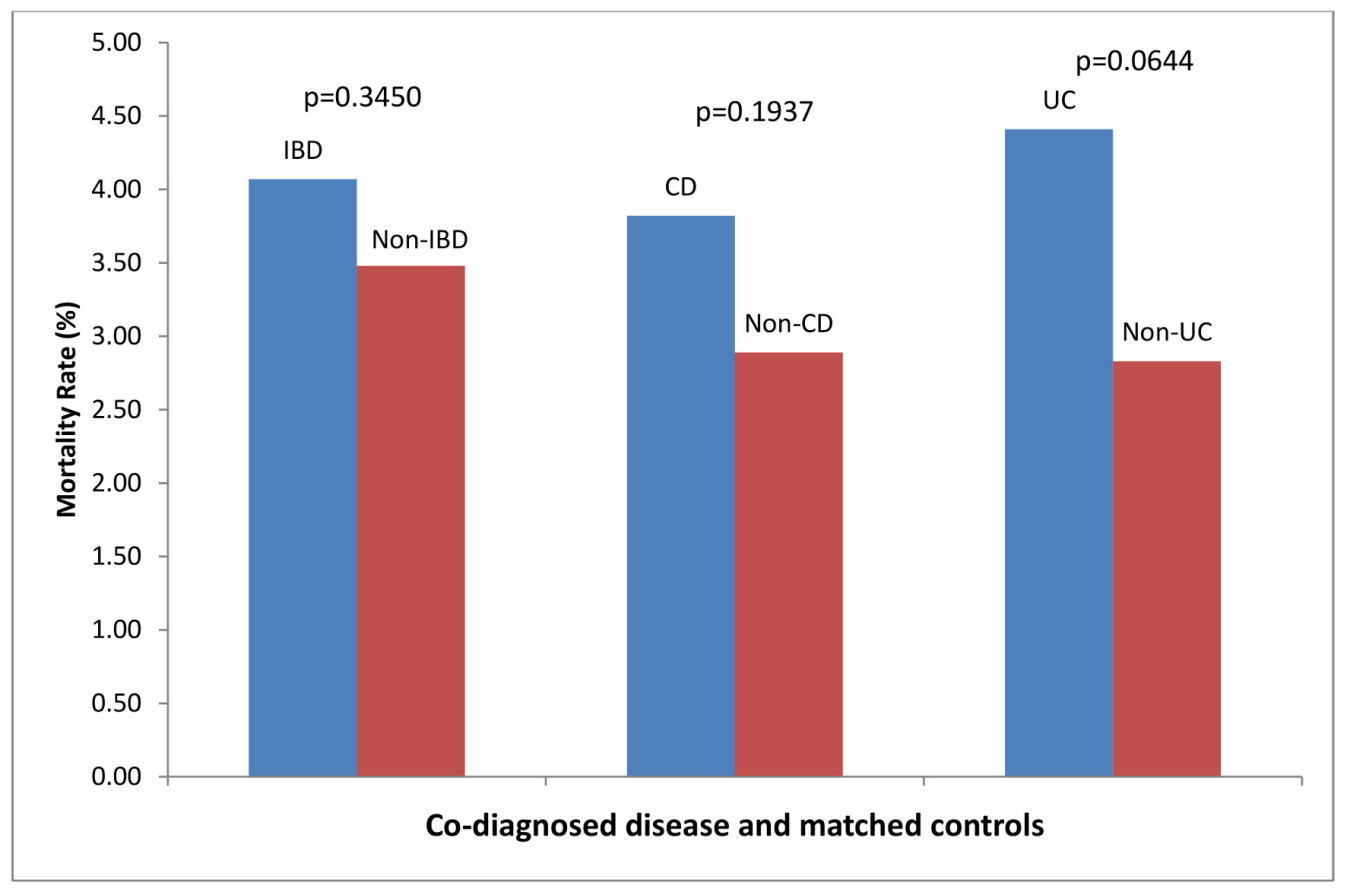
Hospital Mortality Rates for Co-Diagnosis and Matched Controls for Patients Hospitalized with Primary Diagnosis of Congestive Heart Failure (CHF).

### Pneumonia (Fig 3)

There were 5338 patients with a co-diagnosis of IBD that were admitted for PN. These were matched with 10676 controls. 173/5338 (3.24%) patients with IBD and PN died, compared to 413/10676 (3.87%) of controls, p = 0.06. There were 3362 patients with CD admitted for PN. These were matched with 10076 controls. 87/3362 (2.59%) patients with CD and PN died, compared to 428/10076 (4.25%) of controls, p < .0001. This represents a 40% improvement in survival in patients with CD and PN, compared to controls. There were 1982 patients with UC admitted with PN. These were matched with 5954 controls. 86/1982 (4.34%) patients with UC and PN died, compared to 297/5954 (4.99%) of controls, p = 0.24.

**Fig 3:**
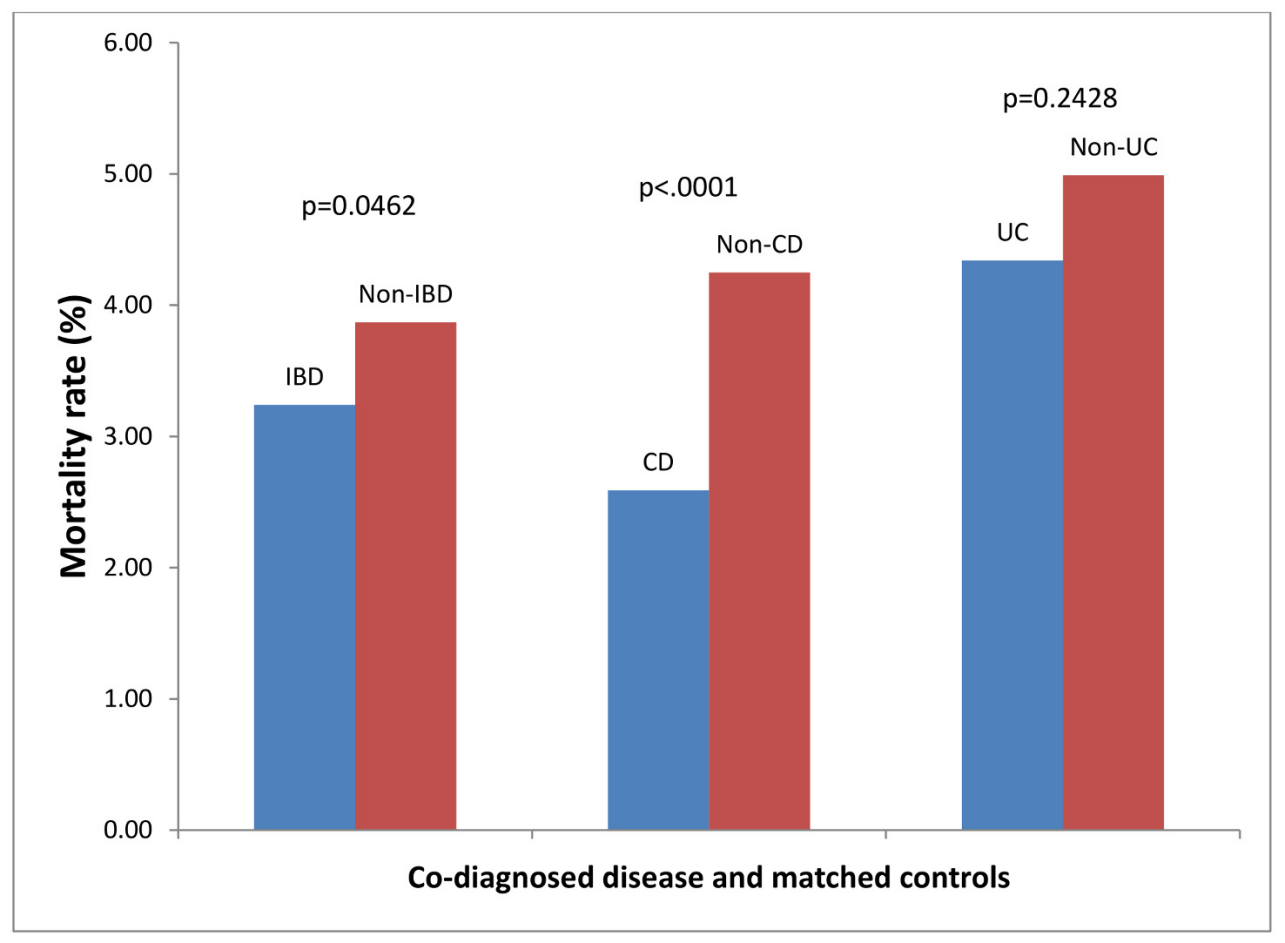
Hospital Mortality Rates for Co-Diagnosis and Matched Controls for Patients Hospitalized with Primary Diagnosis of Pneumonia.

Tables A-C in the S1 File show the associated clinical diagnoses of patients within the various groups, after matching for demographics. These tables show co-diagnoses in patients with UC and CD admitted for pneumonia (PN), acute myocardial infarction and congestive heart failure and their generated control groups.

## Discussion

Comorbidity is generally defined as the presence of pre-existing conditions that increase the likelihood of an adverse outcome. [18] The presence of comorbidities is commonly used to adjust risk-assessment in the analysis of administrative data [5–6]. Despite the assumed negative effects of co-morbidities, some studies have shown a paradoxical improvement associated with co-diagnosed conditions. Some of these types of “paradoxical comorbidities” have been found to positively influence adjusted mortality in patients with AMI and in patients undergoing coronary artery bypass graft (CABG) [7]. This is the first study to show that a co-diagnosis of IBD, CD and UC has a positive impact on mortality in patients that are hospitalized with a primary diagnosis of AMI. In addition, patients with a co-diagnosis of CD have improved mortality when hospitalized for PN. A trend toward worsening mortality was seen in patients with UC that were admitted for CHF.

Patients with inflammatory bowel disease are at higher risk for thromboembolic disorders [10–12]. A recent meta-analysis by Fumery, et al [12] showed an increased risk of venous thromboembolic in IBD patients compared to the general population (RR, 1.96; 95% CI, 1.67–2.30). An increased risk of deep venous thrombosis (RR, 2.42; 95% CI, 1.78–3.30), pulmonary embolism (RR, 2.53; 95% CI, 1.95–3.28), ischemic heart disease (RR, 1.35; 95% CI, 1.19–1.52) and mesenteric ischemia (RR, 3.46; 95% CI, 1.78–6.71) was also found in patients with IBD compared to the general population. Of interest, overall mortality for thromboembolic events in patients with IBD was not found to be increased [11],although previous studies have suggested that the risk of death from venous thromboembolism in patients with IBD was 2.5 times that of the general population [10]. Mechanisms responsible for increased thrombogenesis associated with IBD include acquired factors such as prolonged immobilization and cigarette smoking; direct effects of inflammation on the coagulation cascade, genetic abnormalities associated with IBD, and elevated homocysteine levels [12]. Chronic inflammatory diseases, in general are also associated with an increased risk of AMI [19–20].Despite these factors, an increased risk for AMI in patients with IBD has not been clearly established [21].

Factors that have a negative effect on survival in patients with an AMI include advanced age, female gender, occurrence of pulmonary edema, cardiogenic shock, cardiac arrest or anterior infarction, and the presence of underlying diabetes [22]. The pathophysiologic events following an AMI include local inflammation as well as a systemic inflammatory response. Studies have demonstrated that elevated circulating inflammatory cytokines, chemokines and cell adhesion molecule are components of post-AMI inflammation, while activation of peripheral leukocytes and platelets are also seen. Alteration of immune regulation has been postulated as the cause of these abnormalities [23].In our analysis, compared to controls, there was a 25%, 34% and 24% improvement in survival in patients with IBD, CD and UC prospectively, when they were admitted to the hospital for AMI. We hypothesize that decreased mortality following AMI in patients with IBD occurs by alteration in the upregulated immunity, in part due to these diseases themselves. There is a great interest in the development of anti-inflammatory medications to mediate systemic inflammatory events following AMI [24] Since corticosteroids, immunomodulating medications and/or biologic drugs are administered in a majority of patients with IBD [25], these may have a role in decreasing mortality after AMI in these patients. Alternatively, other unexplained intrinsic factors in patients with IBD may be protective in the setting of AMI. Other possible explanations include a variety of potential selection biases found in administrative data as described below.

Patients with IBD appear to be at increased risk for bacterial and viral pneumonias [13–14]Recently Long et al [26] performed a nested case control study of pneumonia risk in IBD using the IMS Health Inc, LifeLink™ Health Plan Claims Database. They found that the incidence rate ratio (IRR) for PN in patients with IBD was 1.82, (95% CI 1.75–1.88), compared to non-IBD patients. Patients with CD had the highest risk compared to non-IBD patients (IRR 2.05, 95% CI 1.94–2.15). Crohn’s disease was included as a condition with higher risk for pneumococcal disease along with rheumatoid arthritis, systemic lupus erythematosus, neuromuscular diseases and seizure disorders [13]. Patients with inflammatory bowel disease that are treated with immunosuppressive medications, especially corticosteroids, are at higher risk for pneumonia due to opportunistic organisms including Pneumocystis jiroveci pneumonia [15]. Ananthankrishnan, et al [27],suggested that hospitalized patients with IBD and pneumonia had a significantly higher mortality (Odds Ratio for mortality = 3.6, 95% CI 2.9–4.5) compared to those without IBD. Our data shows a non-statistically significant trend toward decreased mortality in all IBD patients that were hospitalized for PN (3.24%) compared to patients without IBD, (3.87%), p = 0.06. In contrast to the study by Ananthakrishnan, we found that a co-diagnosis of Crohn’s disease was protective against mortality in patients admitted for PN. Patients with CD that were admitted for PN had mortality rates of 2.59% compared to a 4.25% mortality rate in controls, (p < .0001). This demonstrates a 40% improvement in survival in patients with CD and PN, compared to controls.

Immunomodulating drugs may have a complex role in patients with Crohn’s disease and infections, both predisposing to infections, but, in some patients, particularly those with CD, preventing subsequent events leading to mortality. In these patients, limitation of inflammatory events following PN, such as direct damage to lung tissue and involvement of other organs and/or the development of multi-organ failure may in part be due to immunomodulating medications. Alternatively, intrinsic alterations of immunity or infection with less aggressive forms of pneumonia may explain decreased mortality in patients with CD. The literature does not contain information that explains why our data shows improved mortality in patients with CD and PN and not in patients with UC, although the UC patients with PN were older than patients with CD and PN. Several potential forms of selection bias that could influence these data are described below. The trend toward higher mortality in patients that were hospitalized for CHF may reflect the expected additive effects of chronic diseases on mortality.

Conditions with a paradoxical relationship to mortality have been noted previously. Jencks, et al [28] found that patients with secondary diagnosis of diabetes mellitus, unspecified anemia, hypertension, angina, ischemic heart disease, and unclassified arrhythmias demonstrated improved 30 days survival following hospitalization. They attributed this occurrence to underreporting of chronic conditions in the higher mortality group. Iezzoni et al. [29]showed that several comorbid conditions including type II diabetes, premature ventricular contractions and angina resulted in decreased mortality after hospitalization for stroke, pneumonia, AMI, or CHF. A common explanation for these occurrences is the presence of selection bias in the admitting and referral process. For example, coronary revascularization was noted to be performed more often in overweight or obese patients than in patients with normal body mass. Decreased overall mortality was also seen in the overweight and obese patient groups [10]. A suggested explanation was that practitioners may pay closer attention to cardiac symptoms in overweight or obese patients, thus increasing their likelihood for referral for procedures. A similar bias could result if clinicians caring for patients with IBD used a higher level of suspicion for the development of AMI, knowing that patients were at higher risk for thromboembolic events. This could then result in earlier hospitalization of patients that were less ill, resulting in improved survival. Similarly, patients on immunomodulator therapy would likely have a lower threshold for reporting symptoms of pneumonia to their clinician, resulting in earlier admission and more rapid administration of antibiotic therapy. Other suggestions for the occurrence of paradoxical co-morbidities include the potential for coding bias. Coding bias transpires when more seriously ill patients admitted for conditions such as AMI or PN have fewer comorbid conditions coded for their hospitalizations. Coding bias also results in additional diagnostic codes for chronic conditions such as IBD included in hospitalized patients who are less ill. By extension, these patients are expected to be found to have a lower mortality in studies that review administrative databases. [29–30].In the present study, it does not appear that coding bias can completely explain the study findings, since a co-diagnosis of IBD, CD or UC did not result in better survival for all three conditions (AMI, CHF and PN). In fact, our study showed that patients with CD or UC both had a trend toward worsened survival when hospitalized for CHF. This would not be expected if coding bias was present.

In summary, having a co-diagnosis of IBD results in improved survival in patients that are hospitalized for acute myocardial infarction, while patients with the co-diagnosis of CD show better survival when hospitalized for pneumonia compared to controls. Although selection and coding biases are considered for these findings of this kind, it is postulated that immunomodulating medications and intrinsic characteristics of patients with IBD are likely contributors to these paradoxical findings.

## Supporting Information

S1 File Tables A-C. (XLSX)
